# Non-target screening in water analysis: recent trends of data evaluation, quality assurance, and their future perspectives

**DOI:** 10.1007/s00216-024-05153-8

**Published:** 2024-02-01

**Authors:** Maryam Vosough, Torsten C. Schmidt, Gerrit Renner

**Affiliations:** 1 Instrumental Analytical Chemistry, University of Duisburg-Essen, Universitätsstr. 5, Essen, 45141 North Rhine-Westphalia Germany; 2 Centre for Water and Environmental Research (ZWU), University of Duisburg-Essen, Universitätsstr. 2, Essen, 45141 North Rhine-Westphalia Germany; 3 Department of Clean Technologies, Chemistry and Chemical Engineering Research Center of Iran, P.O. Box 14335-186, Tehran, Iran; 4 IWW Water Centre, Moritzstr. 26, Mülheim an der Ruhr, 45476 North Rhine-Westphalia Germany

**Keywords:** Non-target screening, High-resolution mass spectrometry, QA/QC in water analysis, Chemometrics/machine learning, Quantitative non-target screening, Aquatic contaminants, Data standardization

## Abstract

This trend article provides an overview of recent advancements in Non-Target Screening (NTS) for water quality assessment, focusing on new methods in data evaluation, qualification, quantification, and quality assurance (QA/QC). It highlights the evolution in NTS data processing, where open-source platforms address challenges in result comparability and data complexity. Advanced chemometrics and machine learning (ML) are pivotal for trend identification and correlation analysis, with a growing emphasis on automated workflows and robust classification models. The article also discusses the rigorous QA/QC measures essential in NTS, such as internal standards, batch effect monitoring, and matrix effect assessment. It examines the progress in quantitative NTS (qNTS), noting advancements in ionization efficiency-based quantification and predictive modeling despite challenges in sample variability and analytical standards. Selected studies illustrate NTS’s role in water analysis, combining high-resolution mass spectrometry with chromatographic techniques for enhanced chemical exposure assessment. The article addresses chemical identification and prioritization challenges, highlighting the integration of database searches and computational tools for efficiency. Finally, the article outlines the future research needs in NTS, including establishing comprehensive guidelines, improving QA/QC measures, and reporting results. It underscores the potential to integrate multivariate chemometrics, AI/ML tools, and multi-way methods into NTS workflows and combine various data sources to understand ecosystem health and protection comprehensively.

## Introduction

Water ecosystems are essential for all living but have endured various anthropogenic threats, ranging from pollution to over-exploitation. As a safeguard, the tools used to assess water quality are crucial. However, the world is rapidly evolving, and the compounds finding their way into water systems are changing. Therefore, continuously adapting modern measurement and data analysis techniques has become desirable and imperative to keep pace. The dominant approaches are targeted screening methods, i.e., target analysis and suspect screening, that have been instrumental in detecting known contaminants [1, 2]. However, their restrictive scope limits their efficacy, potentially allowing unknown or unexpected pollutants to go unnoticed. Non-target screening (NTS), powered by high-resolution mass spectrometry (HRMS), extended those tools, offering a dynamic solution unconstrained by the need to search for specific, pre-identified contaminants [3].

While NTS has ushered in new possibilities for pollutant detection, it has its challenges [4]. Firstly, the sheer volume of data generated by HRMS instruments such as Orbitrap or time-of-flight systems coupled with gas chromatography (GC) or liquid chromatography (LC) can be daunting, demanding sophisticated data management and processing tools. Analyzing and interpreting the vast spectra of chemical signatures also requires much expertise and refined methodologies [5]. Additionally, distinguishing between signal noise, naturally occurring compounds, and emerging pollutants poses another intricate trilemma [6]. The double-edged sword of comprehensive chemical databases is evident in non-target screening. On the one hand, a more extensive database offers more opportunities for compound identification. Conversely, the enormous volume of information can make pinpointing a specific compound akin to finding a needle in a vast haystack. To address this challenge, adopting approaches like PubChemLite can be beneficial, which refines the vastness of the database by focusing on the most relevant and commonly encountered compounds, thus combining the power of comprehensive data with the ease of targeted searches [7]. Beyond these technical hurdles, practical challenges arise regarding standardization across laboratories and regulatory adaptation. As the field of NTS continues to evolve, addressing these hurdles is crucial to harnessing its full potential. In light of the challenges inherent to NTS, the field has witnessed a surge of innovations and methodologies, underscoring the ever-evolving landscape of water quality assessment [3]. These trends promise to refine our understanding and detection capabilities and foster a proactive stance toward potential threats. From advancements in instrument technology, data processing, and method performance assessment to collaborative efforts for database expansion and standardization, these trends represent a renewed commitment to preserving the sanctity of water ecosystems. This Trend article highlights advances in data evaluation approaches, the importance of quality assurance/quality control guidelines and validation protocols for NTS study outputs, and quantitative NTS strategies by focusing on works conducted within the past five years. In this way, different challenges, opportunities, and related concepts to NTS will be explored. In the conclusion section, an outlook for the future trajectory of non-target screening in aquatic ecosystems will be presented based on emerging and expected needs.

## Current data evaluation trends in NTS

The evaluation of HRMS data in NTS consists of two aspects: (1) initial raw spectra processing, aiming to condense via multi-step approaches relevant chemical information from individual measurement data, i.e., feature extraction, and (2) data analysis, putting the chemical information obtained into a meaningful context, e.g., comparing samples for monitoring trend analysis in time series experiments. The outer structure of NTS processing workflows, namely centroiding, peak detection, etc., looks almost harmonized and familiar with target or suspect screenings. However, in detail, the explicit algorithms of the individual steps can differ significantly, making result comparability challenging. In data analysis, advanced multivariate chemometrics methods and machine learning (ML) have become indispensable tools for discovering hidden patterns and identifying correlations where conventional methods are limited. Moreover, high-throughput annotations of unknown features are being performed using open-source molecular discovery workflows that utilize spectral libraries, ML, and cheminformatic tools [8].

Full scan HRMS/MS data storage allows retrospective analysis without re-run sample analyses. This potential has been proposed as an early warning system worldwide to detect emerging pollution threats [9]. In 2019, the NORMAN Digital Sample Freezing Platform (DSFP) was launched to enable digital sample archiving and retrospective suspect screening [10]. However, challenges exist due to the sheer size and complex nature of NTS data, particularly in archiving HRMS data, meta-data, and processing capabilities [11]. As outlined in the following sections, data processing workflows in the NTS generally consist of several steps, from feature extraction to structural elucidation.

### Data processing: feature extraction

Hyphenated HRMS instruments generally produce complex and diverse datasets requiring specific processing and robust processing tools and procedures. Briefly, the main aim of the processing step is to provide a compact “component table” from the original raw data. Processing parameters are crucial to further screening success. The significant points here are extracting highly qualified features, proper feature alignment, redundant feature filtering, and reliable componentization of features to the same unique molecular structure. Recently, different NTS data processing algorithms and methods have been extensively reviewed [6]. Nowadays, in addition to vendor software, open-source tools such as XCMS [12], MZmine [13], SIRIUS [14], MS-DIAL [15], enviMass [16], have been used for HRMS data handling of environmental samples. However, since most of these packages were developed for -omics data, the workflow and parameters are not necessarily appropriate for environmental studies regarding issues like frequent appearances of low-intensity peaks, more highlighted matrix effects, and long-term exposure monitoring. The impact of data preprocessing on the production of different outputs and quality would be another noticeable concern at this stage [68]. This insight requires more thorough research and collaboration to ensure reliable and robust NTS tools and procedures.

The open-source platform PatRoon [17] enables comprehensive environmental NTS data processing, from HRMS pre-treatment to compound annotation. With PatRoon, comparison between algorithms is possible, and feature coverage can be increased by utilizing both common and distinctive features among algorithms. InSpectra, an open-source and modular web-based NTS and suspect screening workflow, was released in 2023 [18]. LC-HRMS data can be archived, shared, and curated through parallel computing with InSpectra. Moreover, InSpectra can identify, track, and prioritize pollution threats in a reproducible and transparent manner.

According to [19], while not limited to water samples, it has been found that vendor software like Thermo Compound Discoverer or Agilent MassHunter is frequently used for LC or GC-HRMS NTS data processing. However, commercial software often presents proprietary constraints, such as limited customization options and exclusive data formats, making transitions or data sharing cumbersome. The closed nature of these tools obscures the understanding of data processing, posing challenges for rigorous scientific evaluations. Furthermore, such software may face interoperability issues due to restrictions on available platforms and variations in tools used by collaborators, hampering seamless teamwork and comprehensive system compatibility. Thus, due to the main disadvantage of commercial software, developing and validating open software that leverages NTS for both GC- and LC-HRMS data while deconvoluting MS2 data for more coverage of chemical exposure in water environments is highly required. An alternative to these “feature profile” (FP) packages is to exploit multi-way chemometric methods for direct “component profile” (CP) production. For example, a set of LC-HRMS measurements can be efficiently modeled by a multi-way algorithm after initial data compression [20]. Multivariate curve resolution-alternating least squares (MCR-ALS) and parallel factor analysis 2 (PARAFAC2) are the most efficient methods. However, other tensor factorization algorithms can also be implemented after extra preprocessing steps. Upon modeling on each data array, three matrices of CPs are generated: resolved “pure” LC profiles, their mass spectral counterpart, and the area under resolved LC profiles as quantification scores. Therefore, data dimensionality can be reduced, decreasing the risk of incomplete componentization and missing compounds that cannot be detected by feature-based peak detection [21]. Data processing protocols based on multiple samples align well with real-world aquatic NTS advancements and perspectives, such as monitoring pollution pathways in river water, wastewater samples undergoing chemical or biological treatment, or water samples measured under a variety of extraction/instrumental conditions [20, 22, 23].

Nevertheless, multi-way methods have prerequisites to consider when applied in GC/LC-MS data processing [24]. As the most flexible algorithm, MCR-ALS provides additional benefits in NTS of water samples with complex GC- or LC-HRMS data structures (e.g., substantial RT shifts and co-elution issues) designed in response to environmental conditions. However, MCR workflows are mainly semi-automated, and solutions may have some ambiguities, though their extent can be mitigated by incorporating sensible ALS optimization constraints. A comparison of FP packages and CP approaches in different water NTS categories would help us understand how they differ qualitatively and quantitatively when extracting a holistic or prioritized subset of chemical spaces. An overall emphasis should be placed on expanding and optimizing fully automated multi-way-based workflows designed to process NTS raw GC- and LC-HRMS data and produce CPs with high completeness and minimal user input.

### Data analysis: pattern recognition

With the “component table” in place, various chemometrics/ML algorithms and visualization tools could be employed for downstream tasks such as uncovering chemical trends and pollution events, monitoring the occurrence and fate of pollutants, assessing of water treatment process, and developing intelligent prioritization criteria to select environmentally relevant features. Hence, it is also crucial to consider data transformations, intensity normalization, and scaling for removing unwanted variations before further analysis. Data processing challenges with NTS include the high dimensionality of HRMS records and the possibility of redundant information, the lack of adequate sample size, and the number of replicates required for statistical testing. An effective data reduction strategy and exploration are therefore required in the first place. In unsupervised NTS, principal component analysis (PCA), hierarchical clustering analysis (HCA), and co-occurrence analysis using Venn diagrams are the most popular methods to evaluate similarities, co-variations, and differences between various NTS cases [5]. While these methods benefit data exploration, one must know their potential and limitations about high dimensional HRMS data [25, 26].

**Fig. 1:**
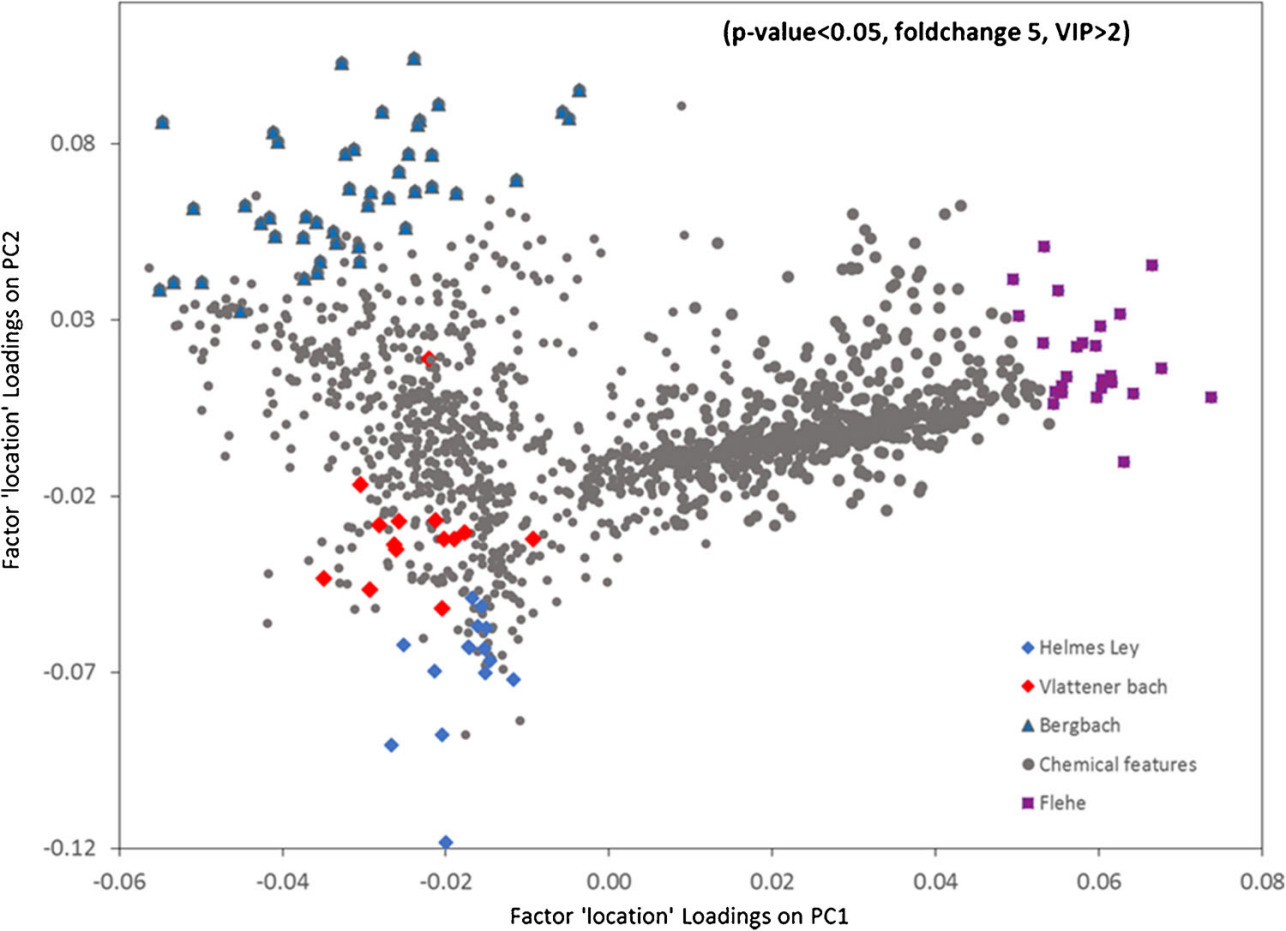
Representation of ASCA(+) loading plot (Factor “sampling location”) to display the final subsets of highly prioritized chemicals (ESI+) strongly associated with different stream water sources by the joint implementation of univariate (volcano test) and multivariate statistical methods (PLS-DA-VIP and ASCA+) [28]. Note: ASCA(+): extension of ASCA (ANOVA Simultaneous Component Analysis) for unbalanced experimental design; PLS-DA-VIP: Partial Least Squares-Discriminant Analysis-Variable Importance in Projection

The recently used supervised classification and multivariate statistical tools in NTS of water samples are partial least squares-discriminant analysis (PLS-DA), its orthogonal directed model (OPLS-DA), support vector machines with linear kernels (SVM), PLS-path modeling, and multivariate ANOVA models [22, 23, 27, 28]. Due to their multivariate advantage, these methods complement univariate statistics (e.g., volcano plots, Mann–Whitney U test, ANOVA) because they consider all features simultaneously and can consider pollutant compound interrelations associated with the outcome (Fig. 1). Multiple validation criteria must be applied to evaluate the performance and generalizability of predictive models following model training, suggesting that procedures for assessing the effectiveness of NTS methods may need to be improved and standardized [6, 29]. However, the previous reports show that NTS data classification can be non-linear in more complex scenarios, such as heterogeneous or highly dynamic behavior of pollution sources or when there is a substantial overlap in the chemical composition of water sources [25, 28]. Therefore, more flexible and robust non-parametric learners such as SVM models with non-linear kernels, artificial neural networks (ANNs), deep learning (DL) algorithms, and ensemble classifiers such as random forest (RF) and gradient boosting algorithms (such as XGBoost and CatBoost) can be exploited in cases where linear classification methods are not robust tools.

Moreover, ML tools have a high potential for use in many other fields within NTS research, including retention time (RT) prediction [30] and collision cross section (CCS) prediction in ion mobility spectrometry (IMS) coupled with HRMS as well [31]. Furthermore, constructing several robust classification models on the same sample set may be necessary to avoid interpretation biases and false discoveries. The final explanatory and predictive outputs would be highly beneficial for future comparison of aquatic ecosystems and uncovering the exposure of relevant pollutants and proper chemical fingerprints in various situations. Such benefits align with what has been emphasized in the literature, which is that NTS data sharing and the development of data repository platforms are essential to facilitate collaborative trials, QA/QC, method developments and comparisons, unify protocols, and contribute to policy and regulatory development.

## Quality assurance/quality control efforts in NTS

The complex nature of NTS requires stringent quality assurance and quality control (QA/QC) measures to ascertain the reliability and validity of results [32, 33]. Many of these QA/QC protocols are general and originate from conventional target analyses, with scarce specific developments for NTS [34]. Therefore, exploring and discussing these measures within the NTS framework and evolving a perspective view on future developments and trends within this field is crucial. In the meaning of QA/QC, there are three layers in the analytical process where quality has to be ensured: Samples, Measurement System, and Data Processing. NTS measurements are organized in long-running batch series, so ensuring stability should be prioritized [33]. It is necessary to ensure sample stability, signifying that samples and their components remain intact and unchanged throughout the analysis. In this situation, one suggestion is to include internal standards (ISs) multiple times within the batch analysis, as Caballero-Casero et al. (2021) put forth [33]. The stability can then be assessed by evaluating the consistency of these internal standards and pooled sample results over time. Next to sample stability, monitoring measurement system stability using quality control charts and batch effect correction is integral for NTS [33, 35]. Including and considering blank samples can be used to identify memory effects at an early stage and prevent cross-contamination.

Moreover, these blank measurements can be considered for later background corrections [34]. Assessing matrix effects is integral to preserving the NTS process’s reliability [33]. Matrix compounds can cause significant shifts in retention time and m/z, with the latter primarily originating from data processing and being associated with increased data dispersion and overlapping effects in the extracted ion traces [6]. QC samples fortified with ISs are vital in assessing matrix effects by comparing QC samples in the absence and presence of respective matrices [33]. Moreover, QC samples provide information about potential interferences, recovery efficiency, error rates for identifications, and precision throughout the analytical process [32, 33, 35]. The number of ISs depends on the complexity of the sample matrices, with each IS requiring successful identification by the workflow’s conclusion [34]. These standards help to normalize data, minimize variability, and augment reliability [36]. Additionally, QC samples are a reference point to validate the outcomes derived from the NTS process [36].

Ensuring that the sample used in the laboratory accurately reflects the real environmental matrix is crucial. It can be done by comparing the standard deviations of sample replicates with those from pooled samples [33, 35]. Pooled samples should also be used for method development to assess and optimize various parameters during sample extraction, data acquisition, and data processing, which is common in metabolomics and can easily be transferred to NTS in water analysis [34, 37]. In the context of QA/QC, quantifying substances identified in NTS accurately presents challenges due to complexities tied to sample matrices, concentrations, and compound characteristics. Although methods like predicted ionization efficiency can be employed to calibrate concentrations against known standards — which will be discussed further in the subsequent section — it should be noted that existing predictive quantification methods in NTS do not meet the same standards as conventional quantification techniques [36, 38]. Predictive quantification in non-target screening involves estimating the quantity of substances based on signal normalization. Due to inherent variations and uncertainties, an acceptable signal enhancement is often set between 30 and 150% of the original signal, depending on the specific context of the analysis. Deviations outside these limits may indicate potential errors or biases in the NTS process [33].

**Fig. 2:**
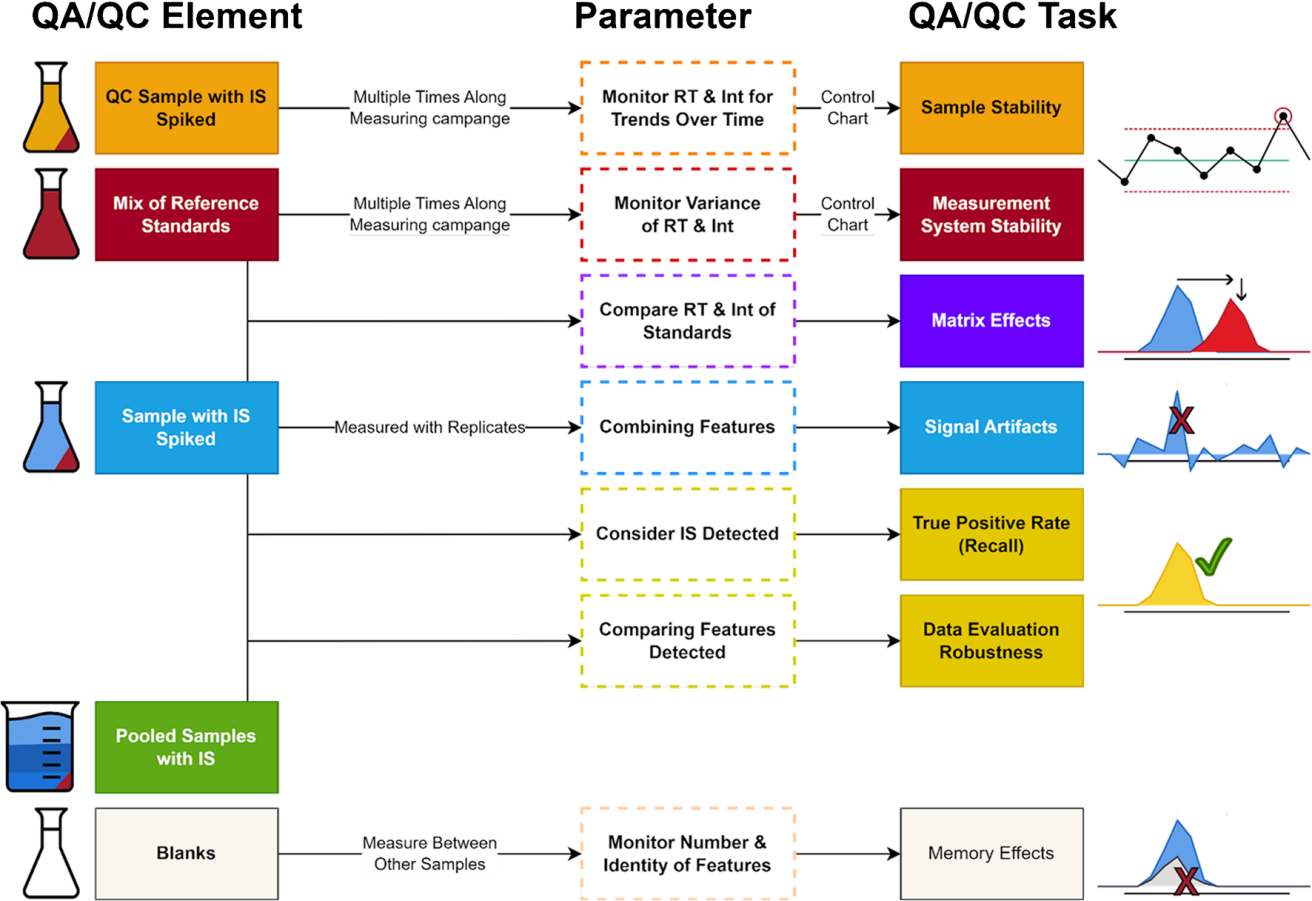
Overview of the essential elements of QA/QC for NTS in water analysis

The advancement and adoption of open-access data processing tools are pivotal for transparent and reproducible NTS results [34]. Reporting data processing and data analysis details is crucial as various algorithms provide significantly different findings, which is still one of the biggest challenges in NTS data processing [6, 20]. However, encouraging interlaboratory comparability investigations can enhance the consistency and comparability of results across different laboratories [33]. Regarding data evaluation, the human element remains a significant unknown. Consequently, it is essential to utilize objective calculation methods, such as those for data similarity considerations, like isotope matching. These methods provide an unbiased evaluation for identification and should be given precedence over subjective approaches such as visual assessments [6, 39]. Clear and detailed guidelines are required for reporting the identification of unknown features and assessing the confidence level [34, 35, 40]. The proposed Identification Points (IP) scoring system by Alygizakis et al. (2023) is based on the already well-established scoring system by Schymanski et al. (2014) [39, 40]. It aims to establish objective standards for non-target screening results, thus improving consistency and quality in data interpretation [39]. In certain instances, penalties can be applied to the IP score, such as when recorded data-dependent scans are lacking or poor fragmentation is observed [39]. An overview in Fig. 2 summarizes the essential elements of QA/QC for NTS.

## Selected NTS approaches in water analysis studies

NTS has been rapidly advancing in recent years since it provides a more holistic view of chemical exposure patterns in different environmental samples by combining HRMS with LC or GC. However, GC-HRMS is still insufficiently utilized compared with LC-HRMS despite the large spectral databases available (e.g., NIST20 and Wiley 11) [5, 19]. Moreover, combined methods have only been used in very few cases [41, 42]. Undoubtedly, further progress would be in developing GC-/LC-HRMS-based NTS workflows for the same aqueous environment. As a result, chemical space coverage can be efficiently increased in various NTS applications, depending on the study design and research questions. The same holds for multidimensional chromatographic systems, HILIC-to-reverse phase chromatography, and combined workflows using different ionization technologies.

NTS workflows have been advancing by adopting passive sampling devices (PSDs) to sample streams and multi-watersheds, which have advantages such as time integrity, cost-effectiveness, fewer disruptions, and greater sensitivity, all of which are beneficial for determining pollution status in streams and determining pollution level [5, 11, 28, 43]. Thus, PSD-based strategies can be exploited to capture both “aggregate” and “cumulative” exposure information about environmental pollution. In summary, using combined strategies in sampling, sample preparation, instrumental measurements, and data acquisition modes, namely data-dependent acquisition (DDA) and data-independent acquisition (DIA) in NTS workflows leading to higher chemical coverage in the aquatic environment, is highly recommended. However, experimental and operational limitations, availability, and complexity of recorded data should be considered [11, 44]. These considerations also apply to the combined target, suspect, and non-target strategies in different NTS scenarios [5, 45]. However, exhaustive elucidations remain challenging and laborious since they need to be confirmed with reference standards and manually verified. Thus, peak prioritization, or specifying the most reliable subset of chemicals based on the research question, is a fundamental part of many NTS studies. Such prioritization ensures that elucidation efforts are focused on the most relevant chemicals and mitigate the risk of overlooking potentially hazardous pollutants. As a result, environmental risk assessment and management efforts could be facilitated using such an approach. There has been a growing number of strategies for prioritizing, whether by effect-directed analysis (EDA), exposure-driven approaches, or their combination [11, 36]. The most recent examples of exposure-driven prioritization strategies are time series LC-HRMS prioritization based on deep learning convolutional neural network (DL-CNN) [46] and group-wise PCA (GPCA) [26], spatiotemporal-based prioritization using PLS-DA-VIP, ASCA(+) [28], binary comparisons using sparse ASCA (GASCA) [22], highly polar pollutant prioritization [47], removal rate ranking in wastewater treatment plants (WWTPs) [48] and source (urban and agricultural) related prioritization [49]. NTS chemical fingerprinting and quantitative source tracking have also been improved in recent years from a method development perspective [50] and in real-world applications, suggesting that a reliable diagnostic subset of features or “smart tracers” can predict pollution sources in watersheds, their relative locations, and quantify processes that are beyond the capabilities of conventional ecosystem sensors through the advancement of ML-based workflows [25]. Alternatively, recent studies suggest that hazard-driven prioritization based on MLinvitroTOX [51], a classifier based on both structural and MS2 data, and online prioritization [52] based on an intelligent MS2 acquisition method could be helpful to focus identification efforts on features with the most significant potential to harm the environment, rather than those that are most abundant.

The use of NTS in advanced oxidation processes (AOPs) is on the rise. It offers several advantages, including the identification of unknown transformation products (TPs), understanding how the water matrix influences the formation of new TPs, determining treatment efficiency on a global scale, detecting abnormalities early, optimizing processes, and making decisions [5, 53]. Furthermore, it was found that AOP treatment evaluation can be improved by adding supercritical fluid chromatography (SFC)-HRMS to the LC-HRMS-based workflow for identifying unknown persistent and mobile organic compounds (PMOCs) found in groundwater samples to achieve greater detection coverage of highly polar compounds [54].

NTS workflows face significant challenges in identifying a prioritized chemical based solely on its accurate mass and MS/MS fragmentation patterns. In particular, for LC-HRMS and with available databases for GC-EI, the assignment of tentative structures in an expert and validated way is still a great challenge and is tedious and time-consuming. Searching in large compound databases (e.g., Massbank, mzCloud, ChemSpider, and PubChem) for possible structures of an elemental formula typically results in numerous hits that need to be ranked and considered further by available MS/MS recorded data, retention time (RT) plausibility, provided meta-data, and limited comparability among instruments, as well. Moreover, developing different in silico tools (Metfrag and CSI: FingerID integrated with SIRIUS) for predicting MS fragmentation patterns and RT predictions using QSPR models are additional validation criteria in NTS workflows and reducing false positive findings. Smaller databases such as STOFF-IDENT [55] and CompTox [56] could facilitate the identification of prioritized unknown chemicals. PubChemLite, a subset of PubChem, was introduced by Schymanski et al. in 2020 for more efficient identification efforts in exposomics and environmental research [7]. Recent progress has been made regarding improving chemical identifications through integrating experimental spectra (ENTACT) into MassBank and developing automated workflows to support non-targeted exposomics and NTS surveys on aquatic samples [57, 58]. A further improvement may be achieved by adding spectral data measured over different types of HRMS systems under varying conditions.

## Quantification in NTS

Quantitative non-target screening (qNTS) is one of the newest analytical fields with substantial challenges, such as the absence of analytical standards, variable ionization efficiency across laboratories and instruments, and the complexity of samples that range from effluents to drinking water [38, 59, 60, 61, 62, 63]. However, recent advancements have brought promising solutions to the forefront. Methods like ionization efficiency-based quantification and machine learning models have improved accuracy [38, 59, 60, 62]. These models, predominantly based on the Random Forest approach, provide reliable predictions and allow for applying different prediction strategies depending on the confidence level of the analytical signal. Additionally, they enable comparability across different laboratories, instruments, and analysis methods [64]. Nevertheless, these advancements have their limitations. Discrepancies in complex matrices, significant errors in modeling approaches, and variability between different instruments and matrices pose challenges [59, 60, 64]. Notably, the need for reference standards for the vast diversity of chemicals can limit quantification accuracy, hindering the progress of non-targeted screening [3]. Looking ahead, the field of NTS is ripe for evolution. The development and improvement of models, especially those utilizing machine learning, are vital future directions. Increasing the range of detectable chemicals and optimizing prediction accuracy could revolutionize the NTS era.

Moreover, there is a pressing need to continue monitoring and managing identified substances. This need will require authorities to control the release of prioritized substances and new chemical candidates, an effort that will undoubtedly shape the future landscape of NTS in aquatic environments. Recent advancements in qNTS emphasize its adaptability and the scientific community’s dedication to refining its methods for more accurate results. From grappling with quantification accuracy to harnessing the power of machine learning, qNTS continues to evolve in response to the challenges it faces. As we look to the future, the field stands ready to embrace new techniques and strategies, all in pursuit of more effective detection, identification, and quantification of unknown chemicals.

**Fig. 3:**
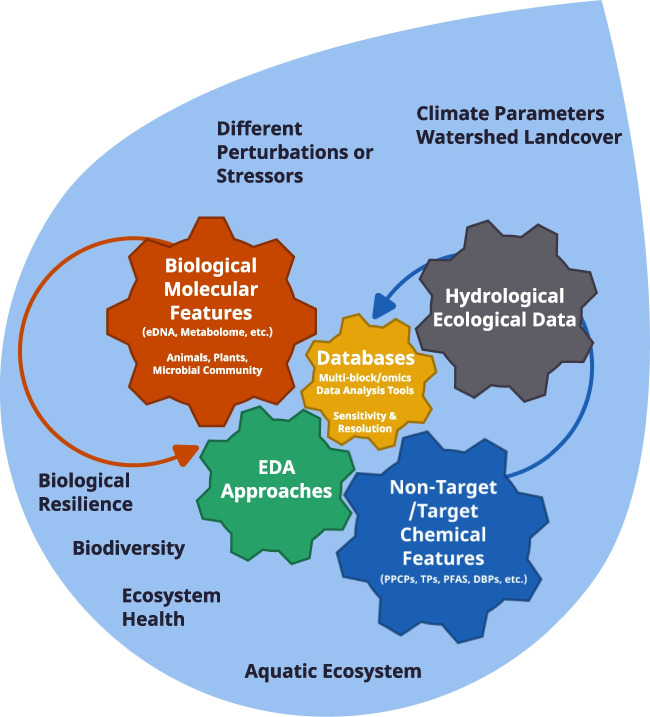
Schematic representation of the emerging NTS-Omics integration framework within an aquatic monitoring and ecosystem health study. Note: PPCPs: Pharmaceuticals and Personal Care Products; TPs: Transformation Products; PFAS: Perfluoroalkyl and Polyfluoroalky Substances; DBP: Disinfection Byproducts; eDNA: environmental DNA

## Conclusion and outlook

Recent advances in NTS of aquatic environments include improved QA/QC guidelines, advanced data processing tools, more comprehensive coverage of “chemical space,” novel prioritization strategies, and increased quantitative approaches. The number of environmental chemicals, suspects, and HRMS spectral databases is growing, and workflows are becoming increasingly automated for chemical identification and structural analysis. Future research needs and challenges related to NTS workflow performance assessment include establishing guidelines for all steps of NTS workflows, improving QA/QC measures, and refining result reporting. In this context, a first step has already been made by esteemed research networks, such as the NORMAN Network, BP4NTA, and the German Water Chemistry Society, who provided guidelines and QA/QC service tools for reporting [3, 4, 66]. Additionally, the initiation of the official norm process for ISO standards marks a significant stride toward standardizing QA/QC in NTS, and its establishment will undoubtedly shape and enhance the practice of QA/QC in NTS in the foreseeable future [67]. The IP scoring system can be further enhanced and adjusted as technological advancements increase data availability. A transition from primarily qualitative to quantitative approaches in NTS could improve the overall quality and accuracy of NTS [36]. QA/QC in NTS data processing must become a focal point for future developments. Currently, valid uncertainty estimation of NTS results is seldom achievable as individual intermediate results are not collected or considered during the complex multi-step NTS data processing [6]. The role of the evaluating analyst in making critical decisions, such as setting input parameters for data processing and analysis, has yet to be studied. Future developments should strive for uniform evaluation criteria, standardized protocols, and the inclusion of statistical methods to describe qualities and uncertainties. From a QA/QC perspective, developing and openly providing reference datasets is crucial, enabling result verification and characterizing strengths, weaknesses, and application ranges of data processing methods.

Different open-source software platforms and statistical packages have been developed and are available for HRMS data preprocessing/processing water samples. Significant progress has been made in integrating multivariate chemometrics methods and AI/ML tools into feature engineering, data formatting, peak annotations, and downstream data analysis, which has led to several opportunities for NTS discoveries in the future. These trends can be expanded to accommodate a variety of statistical approaches, comprehensive LC- and GC-HRMS measurements, and different MS^2^ acquisition modes. Moreover, incorporating the multi-way methods in NTS workflows as an alternative or complementary to the current packages can provide benefits such as providing “pure” qualitative and quantitative information on individual pollutants globally and robustly.

Combination protocols and complementary tools have shown promise in different NTS areas. In this regard, the future expects researchers to conduct multiple robust ML and multivariate/univariate-based prioritizing strategies for selecting the most reliable subset of emerging pollutants based on significant ecological/health risks, environmental exposure/persistence/co-occurrence, or both in several real-world NTS scenarios. Furthermore, retrospective analysis and usage of archived HRMS data will increase due to the increasing trend of sharing data in FAIR repositories. Additionally, an emerging trend will be the integration of chemical NTS data with other data sources in water ecosystems, including Environmental DNA (eDNA) methods (e.g., for microbial communities), -omics-based approaches, ecological and hydrological parameters, and other environmental gradients, using advanced multiblock and statistical analysis tools (Fig. 3). Using integrative approaches allows us to better understand the environment and anthropogenic contaminants’ effect on ecosystem health and ultimately facilitate efforts to protect ecosystems.
